# Long‐Term Follow‐Up of the S‐ICD: A 10‐Years Follow‐Up Study of a Large Single Center Cohort

**DOI:** 10.1111/pace.15173

**Published:** 2025-03-13

**Authors:** Gerrit Frommeyer, Florian Reinke, Benjamin Rath, Julian Wolfes, Kevin Willy, Felix K. Wegner, Julia Köbe, Lars Eckardt

**Affiliations:** ^1^ Clinic for Cardiology II: Electrophysiology University Hospital Münster Münster Germany

**Keywords:** sudden cardidac death, S‐ICD

## Abstract

**Background:**

The subcutaneous implantable defibrillator (S‐ICD) is an alternative to transvenous implantable defibrillators. The present analysis presents real‐world data from patients with S‐ICD and a follow‐up duration of 10 years or more.

**Methods and Results:**

Between July 2010 and November 2013 76 S‐ICD systems were implanted. After a follow‐up duration of 10 years, data from 67 patients (88.1%) was available. Mean follow‐up duration was 10.7 ± 1.3 years. Forty‐seven patients (70.2%) were still alive with active S‐ICD therapy. Eight patients (11.9%) died during follow‐up. In eight patients (11.9%), conversion to a transvenous ICD system was necessary. This was either due to heart failure with indication for biventricular pacing (*n* = 2), bradycardia (*n* = 3), oversensing that could not be solved (*n* = 2), or pocket infection (*n* = 1). In four patients (6%), the S‐ICD system was explanted without replacement for individual reasons. Sixteen patients already underwent two generator replacements, while one generator replacement was performed in the rest of the cohort. Therefore, generator longevity was documented to be within the predicted values. In 10 patients (14.9%), appropriate therapy delivery for ventricular arrhythmias was delivered. In 12 patients (17.9%), inappropriate shock delivery due to oversensing occurred. Of note, this could be resolved in all but two patients. Furthermore, the majority of these episodes occurred in the early years before the implementation of the Smart Pass algorithm.

**Conclusion:**

S‐ICD therapy can be successfully maintained over a long time. Incidence of oversensing significantly decreased with the implementation of novel algorithms and the new S‐ICD generation. However, the present data also points out that in selected individuals conversion to transvenous systems is required.

**Abbreviations**
CM: cardiomyopathy
DFT: defibrillation threshold
ICD: implantable cardioverter/defibrillator
LVEF: left ventricular ejection fraction
S‐ICD: subcutaneous implantable cardioverter/defibrillator
VF: ventricular fibrillation
VT: ventricular tachycardia

## 1 Introduction

The subcutaneous ICD (S‐ICD) has been introduced around 2010 for the prevention of sudden cardiac death (SCD). Since then, the S‐ICD has been shown to be a safe and effective treatment. Initial studies reported positive results with safe and effective prevention of sudden cardiac death. In contrast, transvenous ICD systems are, for example, vulnerable to lead problems that may induce inappropriate therapy deliveries and can also be involved in systemic infections [1, 2]. Early detection problems, including T‐wave oversensing, in particular in patients with channelopathies [3] or hypertrophic cardiomyopathy [4] have been improved by implementing novel detection algorithms. Up to now, the S‐ICD cannot deliver chronic pacing. Therefore, the system is not suitable for patients having or potentially developing a need for bradycardia‐related pacing, anti‐tachycardia pacing (ATP), or cardiac resynchronization therapy (CRT) [5]. Therefore, the S‐ICD has been included in the AHA/ACC/HRS guideline with a class I recommendation for patients at high risk for infections or without adequate venous access [6]. According to the current ESC guidelines the S‐ICD may reduce the risk of lead complications [7].

Though encouraging midterm follow‐up data of the EFFORTLESS registry have been reported long‐term outcomes of S‐ICD technology are still unknown [7]. Nonetheless, registries, as well as randomized studies, have demonstrated clinical effectiveness and safety within usual follow‐up durations. However, there is no data on very long‐term performance of these systems [5, 8–10]. Therefore, the aim of the current study is to provide long‐term follow‐up data in a single‐center cohort including patients with therapy duration of 10 years or more.

## 2 Materials and Methods

The study was conducted in accordance with the guidelines of the Declaration of Helsinki. Between July 2010 and November 2013, a total of 76 S‐ICD systems were implanted at our institution. Patient characteristics are summarized in Table 1. Prior to implantation, S‐ICD screening was performed with a manual screening tool as the automated screening tool was not yet available at that time [11]. Patients were considered eligible for S‐ICD implantation if there was at least one suitable ECG vector. In all patients, an intraoperative defibrillation test was performed. In case of an unsuccessful test, the shock vector was changed to reversed polarity, the shock energy was raised or, if necessary, system components were repositioned intraoperatively under fluoroscopic guidance. For follow‐up, patients were examined at 6 weeks after implantation and every 3–6 months, subsequently. At these times, routine S‐ICD follow‐ups were performed and included evaluation of potential device‐related problems and occurrence of arrhythmias. Adverse events were documented during regular follow‐up in 3–6 months’ intervals.

**TABLE 1:** Baseline characteristics.

Age (years)	41 ± 15 years
Male sex (*n*)	50 (65.7%)
Coronary artery disease (*n*)	7 (9.2%)
Dilated cardiomyopathy (*n*)	13 (17.1%)
Electrical heart disease (*n*)	14 (18.4%)
Hypertrophic cardiomyopathy (*n*)	18 (23.7%)
Congenital heart disease (*n*)	5 (6.6%)
Valvular heart disease (*n*)	5 (6.6%)
Idiopathic ventricular fibrillation (*n*)	6 (7.9%)
Other (*n*)	8 (10.5%)
Primary prevention (*n*)	40 (52.7%)
Secondary prevention (*n*)	36 (47.3%)
LVEF (%)	52 ± 15%

Abbreviation: LVEF, left ventricular ejection fraction.

Data transformation was performed using GraphPad PRISM 6.0 (San Diego, CA, USA) and the SPSS Statistics, version 20.0 (SPSS, Inc., Chicago, IL, USA). Continuous variables are presented as mean and standard deviation (SD), while categorical data are expressed as frequencies.

## 3 Results

Between July 2010 and November 2013 76 S‐ICD systems were implanted at our institution. Forty patients (52.6%) were implanted for primary prevention of sudden cardiac death. Of the 76 patients, 50 were male (65.7%) and 26 female (34.3%). The mean age was 40.6 ± 14.6 years. After a follow‐up duration of 10 years, data from 67 patients (88.1%) was available. The mean follow‐up duration was 10.7 ± 1.3 years.

The mean LVEF was 51.9%. Underlying heart disease included electrical heart disease, hypertrophic cardiomyopathy, ischemic and nonischemic cardiomyopathy, and valvular as well as congenital heart disease.

**FIGURE 1:**
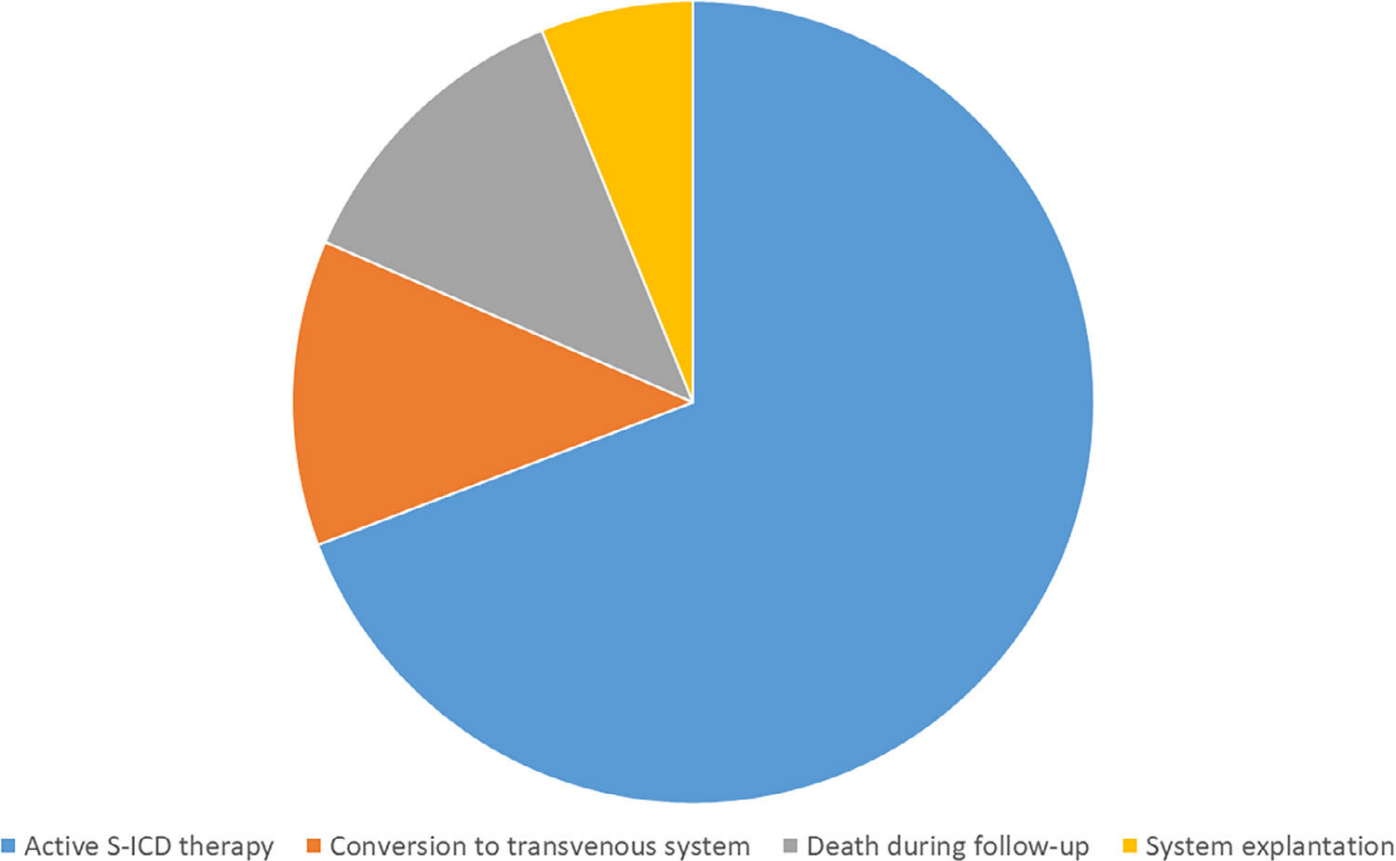
Long‐term outcome after a minimal follow‐up duration of 10 years. [Colour figure can be viewed at wileyonlinelibrary.com]

Forty‐seven patients (70.2%) were still alive with active S‐ICD therapy after a minimal follow‐up duration of 10 years. Eight patients (11.9%) died during follow‐up. Three of these were patients with terminal, severe state of cyanotic congenital heart disease while two died after a longer period of support by a left ventricular assist device (LVAD). One further patient died from a sepsis under immunosuppression, while in the remaining two individuals, the cause of death remains unknown. In eight patients (11.9%), conversion to a transvenous ICD system was necessary. This was either due to heart failure with indication for biventricular pacing (*n* = 2), significant episodes of bradycardia (*n* = 3), oversensing that could not be solved (*n* = 2), or pocket infection (*n* = 1). In four patients (6%), the S‐ICD system was explanted without replacement for different individual reasons (Figure 1).

Sixteen patients already underwent two generator replacements while one generator replacement was performed in the rest of the cohort. Generator longevity therefore was documented to be within the predicted values of the 1st and 2nd generation S‐ICD. During all generator replacements, defibrillation threshold testing (DFT) was performed.

During follow‐up, no events with failure of appropriate shock delivery were reported. In 10 patients (14.9%), appropriate therapy delivery for ventricular arrhythmias was observed. In 12 patients (17.9%), inappropriate shock delivery due to oversensing occurred. Oversensing was mainly due to inappropriate detection of T‐waves or p‐waves. Of note, this could be resolved in all but two patients. Furthermore, the majority of these episodes occurred in the early years before implementation of the Smart Pass algorithm.

## 4 Discussion

The present observational study of a large prospective single center registry demonstrated promising performance of the S‐ICD system during a long‐term follow‐up period of a minimum of 10 years.

Over the last 15 years, the S‐ICD has become an important option for prevention of sudden cardiac death in particular in young patients [12, 13]. In accordance, the current ESC Guidelines for the management of patients with ventricular tachycardia and prevention of SCD have recommended the S‐ICD with a class IIa indication when no pacing is required [6]. In patients with difficult venous access for intravenous lead placement or intracardiac shunts the S‐ICD is listed as an alternative to transvenous systems in the current ESC guidelines, whereas the AHA/ACC/HRS Guidelines give a class I recommendation for these patients [6].

After the initial introduction of the S‐ICD system significant clinical challenges had to be solved. In particular inappropriate shock deliveries which were mostly due to (T‐wave) oversensing occurred and were subsequently reduced by software updates, increased clinical experience in selection of sensing vectors and more extensive operator experience [13, 14, 15]. Up to now, clinical data with follow‐up durations of up to 5 years for the first generation S‐ICD has been reported. For example, the long‐term follow‐up of the EFFORTLESS S‐ICD registry displayed excellent efficacy data on the S‐ICD including a successful conversion rate of ventricular arrhythmias of 98.1% that did not decline during the follow‐up period [13, 16]. Inappropriate shock deliveries were reported in about 17% of patients and were mostly the result of oversensing. The cause of oversensing was heterogeneous. T‐wave and even p‐wave oversensing were the most common causes. In these cases, optimization of sensing vector solved the acute problem. The ongoing optimization of the S‐ICD system including introduction of the SMART PASS algorithm further contributed to the reduction of these events.

In accordance, data of the S‐ICD System PostApproval Study which included 1637 patients showed preserved efficacy of the S‐ICD system and a low incidence of lead‐related complications during a follow‐up duration of 4.2 years [17].

The results of the present study display stable system performance over an extended follow‐up duration of more than 10 years. The mortality rate of 12% as described above can well be explained by the patient characteristics. Three of the deceased patients were individuals with severe cyanotic congenital heart disease and two patients died after a longer period of support by an LVAD.

Conversion to transvenous systems was necessary in 12% of patients. The need of anti‐bradycardia pacing or for cardiac resynchronization therapy for treatment of heart failure represented the most frequent reasons. The necessity of anti‐bradycardia pacing could also have been solved by additional implantation of a transvenous pacemaker as this concept has now been well described in the literature [18, 19]. However, in these individual cases shared‐decision making resulted in explantation of the S‐ICD and consecutive implantation of a transvenous system. In accordance with published data, the occurrence of oversensing was reduced by implementation of software updates and increased experience in choice of sensing vectors. In addition, battery longevity was in the predicted range. During generator replacement DFT was routinely performed. This reflects current clinical practice and was recently confirmed in a study where distinct characteristics of DFT in S‐ICD generator replacement procedures were examined [20].

### 4.1 Limitations of the Study

Due to the nature of the study and the lack of a control group direct comparisons to transvenous ICD systems cannot be drawn. In addition, the heterogeneous patient cohort does not allow any conclusions regarding subgroups. The rare incidence of appropriate ICD interventions furthermore does not allow conclusions regarding potential predictors for the occurrence of arrhythmias.

## 5 Conclusion

The S‐ICD has demonstrated to be a reliable defibrillator system over a period of more than 10 years. S‐ICD therapy can be successfully maintained over a long time. Incidence of oversensing significantly decreased with implementation of novel algorithms and the new S‐ICD generation. However, the present data points out that in selected individuals conversion to transvenous systems can be necessary.

## Conflicts of Interest

G.F., F.R., and L.E. report travel grants and lecture honoraria from Boston Scientific.

## Data Availability

Research data are not shared.
